# Botanical Description of British Plants

**Published:** 1807-10

**Authors:** 


### *Botanical Description of British Plants.*

[ Continued from Vol. xviii. page 270—274. ]

13. Sisymbrium. *S. sophia.*

*Ang.* Flix-weed-water-cress.

*Gen. Desc.* As above.

*Spec. Desc.* *Petals* smaller than the cups. *Leaves* doubly compound, winged. *Pods* long, stiff, crooked. *Seeds* yellow. *Bloss.* yellow. *Walls, rubbish.* Bl. July.

*Use.* This plant has been sometimes prescribed in hysteric and dysenteric cases; and its seeds are given to destroy worms. The pods retain the seeds all the winter, and small birds feed upon them. Cows and sheep eat it; horses and goats are not fond of it; swine refuse it.—*Withering.*

14. Erysimum. *E. officinale.—E. vulgare. Iris sive E. vulgare.*

*Ang.* Hedge mustard, or worm seed. Bank cresses. Scrambling rocket.

*Gen. Desc.* Pod straight, strap-shaped, exactly four-sided. Cal. closed. Summit a knob.

*Spec. Desc.* *Pods* pressed close to the spike stalk, rather conical, hairy. *Leaves* notched, slightly woolly; *lower,* winged; *upper* halberd-shaped. *Stem.* cylind. frequently purple, hairy, branched. *Flowers* in long spikes, terminating, yellow. *Germ.* cylind. tapering upwards. *Sum.* flat, with a shallow nick. *Seeds* oblong.—*Road sides, rubbish, &c.* Bl. May, June.

*Use.* The taste of this herb is somewhat acrid, especially the tops of the flower-spikes. Its seeds are considerably pungent, and appear to be nearly of the same quality with those of the mustard, though weaker. This plant is said to be attenuant, expectorant, and diuretic; and has been strongly recommended in chronical coughs and hoarsenesses. It is mentioned by Rondeletius, that a hoarseness, occasioned by loud speaking, was cured in three days by means of it: other medical writers also have borne testimony to its good effects in this disorder, and Dr. Cullen for that purpose recommends the juice mixed with an equal quantity of honey or sugar: in this also it is a useful remedy in ulcerations of the mouth and throat. In most cases of disease, perhaps its seeds, as more pungent, should be preferred to its leaves.—*Woodville.* "Hedge mustard is beyond any thing in ulcers of the throat; this was found by experience by the Hon. Harry Gray, Esq. when all advice of Doctors and Surgeons availed nothing: this from his own mouth." *MS. note in a copy of Parkinson, which formerly belonged to Mr. Saunders, surgeon at Stourbridge.* It is warm and acrid to the taste; and, when cultivated, is used as a spring pot herb. Birds are fond of the seeds. Sheep and goats eat it; cows, horses, and swine refuse it. *Withering.*

15. Erysimum. *E. barbarea.*

*Ang.* Winter cresses. Winter rocket. Rocket worm seed.

*Gen. Desc.* As above.

*Spec. Desc.* *Pods* indistinctly four-cornered, long, slender, slightly flatted. *Leaves* lyre shaped, terminating segment circular, half embracing the stem. *Stems* strong, eight-furrowed. *Bloss.* yellow.—*Walls, river banks, watery places.* Bl. May, October.

*Use.*

*Use.* The leaves are used by the common people in Sweden in sallads, early in the spring and late in the autumn: they are there also boiled as kale: and is sown in gardens for an early spring sallad.—*Linn.* In England also it is cultivated for the same purpose by the name of French cress. Cows eat it; sheep and goats are not fond of it; horses and swine refuse it.—*Withering.*

16. Erysimum. *E. alliaria.*—*Alliaria.*

*Ang.* Jack-by-the-hedge. Sauce alone. Garlic worm seed. Stinking hedge mustard.

*Gen. Desc.* As above.

*Spec. Desc.* *Leaves* heart-shaped, bluntly and irregularly serrated, alternate, on leaf stalks. *Stem.* cylind. *Flowers* in a corymbus. *Ditch banks, shady places.* Bl. May.

*Use.* The leaves have moderate acrimony, and a strong flavor resembling that of garlic or onions; they give the same kind of taint to the breath as those roots, and have been used for the same culinary purposes: on drying, the sensible qualities are lost. Its medicinal character is that of a powerful diaphoretic, diuretic, and antiscorbutic; it has been deemed useful as an expectorant and deobstruent, in humoral asthmas, and other cases of dyspnœa. It has also been much esteemed as an external application, to promote suppuration. Boerhaave (*Hist. Plant. Lugd. Bat.* 437) cured a gangrene of the leg, arising from a neglected fracture and contusion, by applying the bruised leaves with wine.—*Woodville.* An outward application of the leaves has been recommended in gangrenous and cancerous ulcers.—*Lightfoot.* The seeds excite sneezing.—The leaves are eaten by the Prussians along with salted meats in the spring; they are useful with lettuce, and the colder sallads. In Wales it is much used as a frying herb. When it grows in poultry yards, the fowls eat it, and it gives an intolerable rank taste to their flesh.—Cows and goats eat it; horses, sheep, and swine refuse it.—*Withering.*

17. Erysimum. *E. cheiranthoides.*

*Ang.* Treacle wormseed.

*Gen. Desc.* As above.

*Spec. Desc.* *Stem* v. much branched, 1 to 2 cubits high, rough, stiff, straight, scored. *Leaves* spear-shaped, oblique, waved and toothed, entire, roughish. *Pods* expanding quadrangular. *Seeds* oblong, yellow brown, intensely bitter. *Bloss.* small, yellow. *Sum.* small, scarcely divided. *Osier-holts, river banks, corn fields.* Bl. July.

*Use.*

*Use.* The seeds are used by country people to destroy worms; and with good effect.—Horses, cows, sheep, goats, and swine eat it.—*Withering.*

18. Brassica. *B. napus.*

*Ang.* Wild navew. Rape. Nape. Rape cabbage.

*Gen. Desc.* Cal. upright, close. Glands, one between each shorter stamen and the pistil, and 1 between each pair of longer stamens and the calyx. Seeds globular.

*Spec. Desc.* *Root* a regular continuation of the stem; spindle shaped. *Root-leaves* lyre-shaped, smooth; *stem-leaves* heart-shaped, oblong, embracing, toothed, smooth, sea green. *Stem* branched, about 2 feet high, smooth, *Cal.* expanding, yellow green. *Summit* a flatted knob. *Pod* often with three or four warty excrescences. *Bloss.* yellow.—*Ditch banks, corn fields.* Bl. May.

*Use.* The roots of the cultivated variety may be eaten like turnips, but they have a stronger taste: its seeds, which are called cole seed, afford a large quantity of expressed oil, which is known by the name of *Rape oil.* What remains after the expressing of the oil, is called *Oil cake,* and is used for fattening oxen. In Norfolk these cakes are used as manure, broken in pieces, and strewed upon the land; it is thought to be very efficacious, and is sold for 4l. to 6l. per ton; half a ton is laid on an acre.—*Woodward.* Cows, goats, and swine eat it.—*Withering.*

19. Brassica. *B. rapa.*

*Ang.* Turnip-cabbage. *Var.* Turnips. Knolles.

*Gen. Desc.* As above.

*Spec. Desc.* The *Root* a regular continuation of the stem, round, depressed, fleshy, (in *var.* oblong), *root-leaves* rough, indented. *Cal.* and *Bloss.* yellow.—*Corn fields.* Bl. April.

*Use.* The roots are either eaten raw, boiled, or roasted; pepper is commonly used with them. They relax the bowels, and are supposed to sweeten the blood; they are hurtful to pregnant women, or to hysterical persons, and those who are subject to flatulencies.—The juice well fermented, affords by distillation an ardent spirit. The rind is acrimonious. The roots kept in sand or in a cellar during the winter, send out white shoots and yellowish leaves, which being rather sweet and not unpleasant to the palate, are used as sallad, when other esculent herbs are not to be had. But the greatest use of turnips is in feeding oxen and sheep in the winter.—*Withering.*

20. Brassica. *B. oleracea.*

*Ang.*

*Ang.* Sea cole wort. Sea cabbage. Common cabbage.

*Gen. Desc.* As above.

*Spec. Desc.* *Root* a regular continuation of the stem, cylind. fleshy. *Stem-leaves* much waved, variously indented, sea-green, often with a mixture of purple; the *lower* egg-shaped, sitting; the *upper* mostly strap-shaped. *Cal. leaves* egg-shaped, broad, yellow. *Flowers* large, yellow. *Pods* short, swelling. *Seeds* dusky purple.—*Cliffs on sea-coast.*

*Use.* Early in the spring, the sea cabbage is preferred to the cultivated kinds, but, when gathered on the sea coast, it must be boiled in two waters, to take away the saltness. The roots may be eaten like those of the preceding species, but they are not so tender. All the different varieties of the cultivated garden cabbage, which are much in use at our tables, originate from this. The red cabbage is chiefly used for pickling; in some countries the white cabbage, when full grown in autumn, is buried, and thus preserved through the winter. It is a common practice in Germany to cut them to pieces, and along with some aromatic herbs and salt, to press them close down in a tub, where they soon ferment; they are then eaten under the name of sour crout. Cabbage is apt to be infected early in the year with an insect called the Chrysomela saltatoria, and afterwards the Papilio Brassica; it may be preserved from the former, by strewing the ground with soot; and from the other by whipping the plant with the green boughs of alder. Cabbages sowed or planted for several years together in the same soil, become smaller in the head, and the roots knotty; which is occasioned by the larvæ of flies.—Cows grow fat upon the leaves.—*Withering.*

21. SINAPIS. *S. arvensis.*

*Ang.* Chadlock. Wild mustard, or charlock. Corncale.

*Gen. Desc.* Cal. expanding. Bloss. claws upright. Glands between the shorter stamens and the pistil, and between the longer stamens and the calyx. Pod beaked, opening valves shorter than the partition.

*Spec. Desc.* *Pods* with many angles, swoln and bunched out by the seeds, smooth, longer than the two-edged beak. *Cal. leaves* slightly compressed, spreading, yellow. *Stem* scored, reddish, hairy. *Leaves* harsh, deeply indented and serrated. *Petals* yellow. *Seeds* brown.—*Cornfields.* BL. May.

*Use.* In Scandinavia this plant is boiled and eaten as cabbage:

cabbage: and in Ireland the tender tops are collected for the same purpose. Cows, goats, and swine eat it; sheep are very fond of it; horses generally refuse it.—*Withering.*

22. SINAPIS. *S. nigra.*—*S. sativum.* *S. rapifolis.*

*Ang.* Common mustard. Black mustard.

*Gen. Desc.* As above.

*Spec. Desc.* *Pods* smooth, laid flat to the spike-stalk, short, parallel; sometimes slightly hairy, but the *beak* smooth. *Stem* branched, cylind. scored, upper part smooth. *Root-leaves* rough. *Stem-l.* smooth; the *upper* frequently simple, spear-shaped, toothed. *Fruit-st.* short. *Cups* and *Bloss.* yellow.—*Corn-fields, road sides.* BL. June.

*Use.* The seeds of this plant, and those of the S. alba, (which are preferred by the Edinb. Coll.) manifest no remarkable difference to the taste, nor in their general effects, and therefore answer equally well, both for the uses of the table and the purposes of medicine; they have an acrid pungent taste; and when bruised, this pungency shews its volatility by powerfully affecting the organs of smell. Mustard is considered to promote appetite, assist digestion, attenuate viscid juices, and by stimulating the fibres, to prove a general remedy in paralytic and rheumatic affections. Taken in considerable quantity, it is found cathartic and diuretic, and therefore useful in dropsy. "An ordinary table spoonful of the unbruised seeds is not heating to the stomach, but stimulates the intestinal canal, and commonly proves laxative." *Cullen ii.* 171. It has been recommended as an antiscorbutic: the seeds bruised and mixed with white wine, cured an inveterate and fatal scurvy which had arisen during the confinement of a siege. *(See Ray Hist. p.* 603.*)* Though it is said by Haller, that the use of mustard disposes the humours to putrescency and injures the stomach; *(See Hist. Stirp. Helv. n.* 465,*)* an opinion into he was probably led by the erroneous supposition that it contained volatile alkali: *(See Cullen, l. c.)* The great pungency is to be ascribed not to volatile alkali, but to the essential oil contained. Bergius found mustard of great use in vernal intermittents; a spoonful of the whole seeds, taken three or four times a-day, during the apyrexia; when the disease was obstinate, he added flower of mustard to the bark. Externally the seeds are used as a stimulant, or sinapism; when moistened with vinegar and kept for a day, the powder becomes considerably more acrid, which should be attended to in its external use.—*(Cullen.)* It may most conveniently veniently be given unbruised, to the quantity of a spoonful, or half an ounce to a dose.—*Woodville.* The seeds reduced to powder, make the common mustard so much in request at our tables; they yield a considerable quantity of expressed oil, which partakes but little of the acrimony of the plant. Taken inwardly in the quantity of a meat spoonful or more, they gently loosen the bowels, and are of service in asthma, chronic rheumatism, and palsy. The powdered seeds curdle milk, and give a strong impregnation to boiling water; this infusion taken in a considerable quantity is emetic; in smaller doses, an useful aperient and diuretic. Cataplasms formed with the powdered seeds, crumb of bread and vinegar, are very commonly applied to the soles of the feet, as stimulants, in fevers which require such treatment; they are used with advantage, topically applied in fixed rheumatic and sciatic pains. Wherever a strong stimulus is required, that acts upon the nervous system, without exciting much heat, there is none preferable to mustard seed.—*Withering.*

*Note.* The S. alba, or white mustard, *with rough pods,* is commonly sown for early sallading. The seeds have the same properties as the above.

23. Raphanus. *R. raphinistrum.*

*Ang.* White flowered charlock. Wild radish.

*Gen. Desc.* Cal. close, upright. Nect. glands two between the shorter stamens and the pistil, two between the longer stamens and the calyx. Pod round, but protuberating, with cells, and nearly jointed.

*Spec. Desc.* *Pods* round, jointed, smooth, one cell, joints falling off separately. *Stem* rough, sea green, branched from the bottom. *Leaves* sometimes hairy; *lower* lyre-shaped, wings alternate, heart oblong, lowermost very small, odd one large, rounded, scolloped; *upper* oblong-spear-shaped, scollop-serrated. *Leaf-stalks* and *calyx* hairy. *Petals* yellow, white, violet, or pale red, always with dark veins.—*Among corn.* Bl. June, July.

*Use.* In wet seasons it grows in great quantity amongst the barley in Sweden, and it deserves to be remarked, that in those provinces, and in those seasons wherein this plant abounds, the common people, who eat barley bread, are afflicted with very violent convulsive complaints. *Aman. acad. vi.* 430.—Horses eat it; cows refuse it.—*Withering.*

Class

### CLASS XVI. MONADELPHIA.

### Monadelphia. Triandria.

1. JUNIPERUS. *J. communis. J. vulgaris.*

*Ang.* Juniper tree.

*Gen. Desc.* Diæcious. M. Cal. a scale of the catkin. Bloss. 0. Fem. Cal. 3-div. Petals 3. *Pistils* 3, drupa juicy, closed, one-celled, many-seeded; with three tubercles formerly the calyx.

*Spec. Desc.* *Leaves* three together, expanding; sharp pointed, longer than the berry. *Berry* continuing 2 years green, at length blackish purple. *Anthers* under the lateral scales often five. *Bark* reddish.—*Heaths.* BL. May.

*Use.* Both the tops and berries are directed for use in our Pharmacopœias, but the latter are usually preferred; and are generally imported from Holland and Italy. The berries have a moderately strong not disagreable smell, and a warm pungent sweetish taste, which is followed, when long chewed, by considerable bitterness: the sweetness resides in the juice or soft pulpy part of the berry; the bitterness in the seeds; and the aromatic flavor in oily vesicles, spread throughout the substance both of the pulp and the seeds, and distinguishable even by the eye. *See Lewis M. M. p.* 362. They are chiefly used for their diuretic effects; they are also considered to be diaphoretic, stomachic, and carminative. Of the efficacy of juniper berries in many hydropical affections, there are various relations by physicians of great authority, as Du Verney, Hoffman, Boerhaave, Van Swieten, &c. but authors are not agreed which preparation is the most efficacious. *See Cullen M. M. ii. p.* 187, *Hoffman, &c.* But as they are now seldom relied on for the cure of dropsy, and only called to the aid of more powerful remedies, it is justly observed by a modern author, *(Duncan New Ed. Disp.)* " that, perhaps, one of the best forms under which the berries can be used is a simple infusion; this either by itself, or with the addition of a little gin, is a very useful drink for hydropic patients." Medical writers have also spoken of the utility of Juniper in nephritic cases, uterine obstructions, scorbutic affections, and some cutaneous diseases; and in the two last mentioned complaints, the tops and wood are said to have been employed with more advantage than the berries. Of the wood and tops the virtue is described by Bergius, as diuretica, sudorifica, mundificans; of the berries, diuretica, nutricus, diaphoretica.

Linnæus

Linnæus tells us, that the Laplanders drink infusions of these berries, as the English drink tea and coffee; and that the Swedes prepare a beer from them, which is in great estimation for its diuretic and antiscorbutic qualities—*Woodville.* The berries likewise afford a pleasant wine.—*Du Hamel.* The berries, which are two years in ripening, afford, when bruised, a pleasant diuretic liquor, but it is not easy to prevent it from turning sour: it is esteemed a good antiscorbutic. The Swedes prepare from the berries an extract, which by some is eaten for breakfast, but it is fitter for a medicine than for food. The spirit impregnated with the essential oil of these berries is every where known by the name of Gin, Geneva, or Juniper water; but there is great reason to believe that the gin usually sold in England, instead of the genuine Geneva imported from Holland, is nothing but the common fermentacious spirit, imbued with turpentine or other materials to give it a flavour. Gum Sandarach, commonly known by the name of Pounce, is the product of this tree, exuding through the crevices of the bark, or the perforations made by insects. The wood is hard and durable: the bark may be manufactured into ropes.—It grows in fertile or in barren soils; on hills or in valleys; in open sandy plains or in moist close woods: its trunk on the sides of hills grows long, but, on rocky mountain tops and on bogs, it is little better than a shrub. It is easily transplanted and bears cropping, but the grass will not grow beneath it; the avena pratensis destroys it. Horses, sheep, and goats eat it.—*Withering.*

( To be continued. )

